# Leveraging International Health Regulations (2005) to enhance health security in the Eastern Mediterranean Region: a retrospective analysis from 2016 to 2023

**DOI:** 10.1136/bmjph-2024-001048

**Published:** 2025-01-09

**Authors:** Mohamed Elhakim, Mahgoub Hamid, Amgad Elkholy, Dalia Samhouri

**Affiliations:** 1 WHO Health Emergencies Program, World Health Organisation Regional Office for the Eastern Mediterranean, Cairo, Egypt

**Keywords:** Emergencies, Public Health, Community Health

## Abstract

**Introduction:**

The WHO Eastern Mediterranean Region (EMR), consisting of 22 member states, faces significant health security challenges exacerbated by socioeconomic, political and environmental factors. This study aims to assess the efforts to enhance health security in the EMR from 2016 to 2023 through the implementation of the International Health Regulations (IHR) (2005) core capacities.

**Methods:**

A narrative review was conducted using IHR Monitoring and Evaluation Framework (MEF) tools, such as the State Party Self-Assessment Annual Report (SPAR) and the Joint External Evaluation (JEE), to evaluate the region’s preparedness and response capabilities. Other assessments, such as the Universal Health and Preparedness Review (UHPR), after-action and intra-action reviews and simulation exercises, were included.

**Results:**

The evaluations identified critical gaps in health security infrastructure, highlighting the need for comprehensive strategies and external support, including the Pandemic Fund and National Action Plans for Health Security. While progress has been made, challenges remain due to emerging and re-emerging diseases and regional humanitarian crises.

**Conclusion:**

Despite improvements, the EMR continues to face significant health security challenges. Increased advocacy, capacity building and multisectoral collaboration, particularly through the One Health approach, are essential for future preparedness.

**WHAT IS ALREADY KNOWN ON THIS TOPIC**

- The health security challenges in the Eastern Mediterranean Region are beyond any country’s capacity to respond alone without the need for support from WHO. The International Health Regulations (IHR) (2005) offer an opportunity for strengthening regional preparedness and response capacities, but consistent implementation across the region remains a challenge.

**WHAT THIS STUDY ADDS**

- This study provides an in-depth evaluation of the implementation of IHR core capacities in Eastern Mediterranean Region from 2016 to 2023, using IHR Monitoring and Evaluation Framework (MEF) tools such as the Joint External Evaluation and the State Party Self-Assessment Annual Report. The findings highlight key gaps in capacities that hinder health security efforts, as well as successful initiatives, including the National Action Plan for Health Security and the role of the Pandemic Fund in enhancing health security.

**HOW THIS STUDY MIGHT AFFECT RESEARCH, PRACTICE OR POLICY**

- The study underscores the importance of a multisectoral approach and international collaboration, particularly the One Health approach, in addressing health security gaps in the EMR. The insights provided by our study will inform future efforts to strengthen preparedness and response, guiding policy reforms and investment in health systems to better mitigate public health threats.

## Introduction

 The Eastern Mediterranean Region (EMR) of the WHO has a unique geopolitical context with a huge diversity being composed of 22 member states,^1^ spread along the biggest two continents globally, Africa and Asia, with seven member states in Africa and 15 in Asia.^2^ This geographically diverse region has historically grappled with a complex interplay of socioeconomic, political and environmental factors that have given rise to health vulnerabilities,^3 4^ with low and lower-middle-income countries representing 13 out of 22 in the region (59.1%).^5^

The EMR has been confronted with multiple health challenges that have not only affected its healthcare systems but also tested the resilience of its member states. Over the years, the region witnessed the emergence of novel health threats alongside the persistence of existing ones.^6^ The burden of communicable diseases, non-communicable diseases and humanitarian crises has imposed significant strains on healthcare infrastructures, underscoring the urgent need for cohesive strategies to strengthen health security.^7^

The health security, represented by the capacity to prevent, detect, respond to and recover from health crises, and defined as the activities required, both proactive and reactive, to minimise the danger and impact of acute public health events that endanger people’s health across geographical regions and international boundaries, according to WHO’s definition,^8^ has emerged as a predominant concern in the EMR. Recently, a significant increase in the number of emergencies in the region was noticed due to multiple hazards. More than 50 disease outbreaks were responded to in the EMR in 2022, compared with 31 in 2021 and 14 in 2020, and the number of people in need of humanitarian assistance has increased from 102.3 million to 127.3 million.^9^

Lack of security in the majority of the EMR countries, major natural disasters, including the floods in Pakistan,^10^ food insecurity in Somalia,^11^ earthquake in the Syrian Arab Republic^12^ and drought in the Greater Horn of Africa,^13^ aggravated by the COVID-19 pandemic and severe economic crisis in some member states including Lebanon and the Syrian Arab Republic,^14^ have contributed to an unprecedented level of humanitarian need and vulnerability across the region.^9^ Several member states in the EMR are currently facing protracted humanitarian crisis, including Afghanistan, Palestine, Somalia and Sudan, with multiple disease outbreaks, natural disasters, armed conflicts and internal and external displacement throughout the past years.^15^,^18^

The political instability and insecurity contribute to hindering the ability of member states and WHO to effectively respond to different health emergencies. The healthcare facilities are widely attacked across the region. Around 245 attacks on healthcare facilities were reported in 2022 in seven member states, namely, Afghanistan, Libya, Palestine, Somalia, Sudan, the Syrian Arab Republic and Yemen, resulting in 80 attacked fatalities and 150 injuries among healthcare workers and patients, according to the Surveillance System for Attacks on Healthcare^19^ Thus, the EMR reported about 37% of deaths and 35% of injuries due to such attacks worldwide.^9^

**Figure 1:**
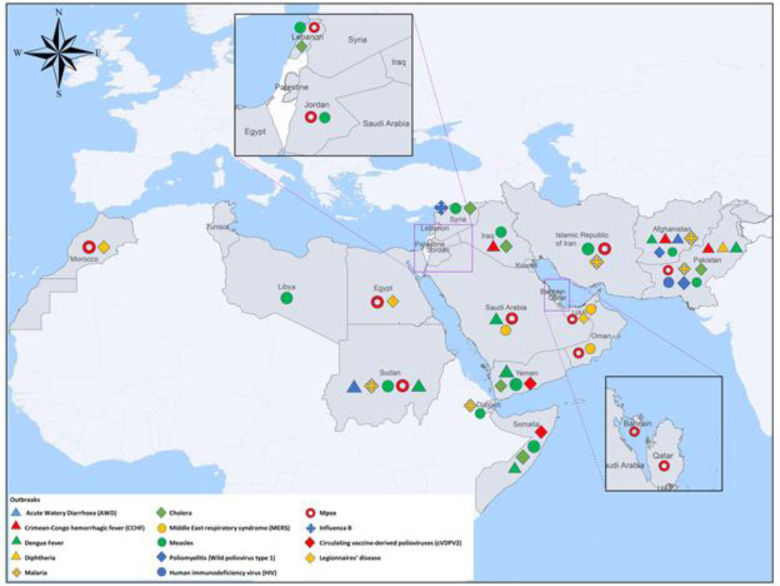
Map of outbreaks reported in the WHO Eastern Mediterranean region, up to 15 December 2023 (WHO website: https://www.emro.who.int/pandemic-epidemic-diseases/outbreaks/index.html).

The EMR was also home for emerging diseases, including the Middle East Respiratory Syndrome (MERS), a viral respiratory disease caused by MERS coronavirus, reported for the first time in Jeddah, Saudi Arabia, in June 2012.^20^ The region also suffered from the global emergence of the COVID-19 pandemic with the first case reported in the region, in the UAE, on 29 January 2020. Since then up to 30 October 2023, the EMR reported 23 403 218 confirmed cases with 351 721 deaths, for a (CFR) of 1.5%.^21^ Emerging, and re-emerging diseases, including vaccine-preventable diseases, represent a huge burden on the health systems of the EMR member states, including Crimean Congo haemorrhagic fever, chikungunya, cholera, diphtheria, influenza H5N1, leishmaniasis, measles, plague, wild poliovirus and Q fever^22^ (figure 1).

In addition, the porous borders between the member states have further amplified the potential for cross-border transmission of infectious diseases on the human and animal sides, necessitating collaborative efforts to monitor, detect and respond to outbreaks effectively.^23^ Therefore, in order to face health challenges that emerge and re-emerge at different levels, human-animal-environment, efficient collaboration, coordination, risk communication and community engagement (RCCE), and concerted action between different sectors are highly needed, in addition to the governance and institutionalisation of the One Health approach.^24^

Hence, the EMR member states realised the importance of being better prepared to prevent public health emergencies and to detect and respond to public health threats in order to stop the devastating impact these threats have on people’s lives and well-being, as well as on travel and trade, national economies and the society as a whole.^25^ Public health challenges are very complex and a holistic, multisectoral and multidisciplinary approach is needed for addressing the health gaps and advancing the coordination for health emergency preparedness and health security, and it is essential for the implementation of the International Health Regulations (IHR, 2005) core capacities.^26^

The aim of this narrative study is to demonstrate the effort spent in the WHO EMR, to leverage the health security following the IHR core capacities and how member states in the region are using the assessments and evaluations to determine the IHR core capacities implementation through the IHR Monitoring and Evaluation Framework (MEF) and other WHO tools.

## Methods

The IHR MEF, developed by the WHO, supported member states through different approaches, using capacity assessment tools, including the IHR SPAR and the JEE, helping to find the existence of the national capacity and functional assessment tools, including the after-action review (AAR)/intra-action review (IAR), and the simulation exercises, helping to assess the functionality of the capacity. It ensures the mutual accountability of IHR States Parties and the Secretariat for global public health security through transparent reporting and dialogue.^27^

**Figure 2:**
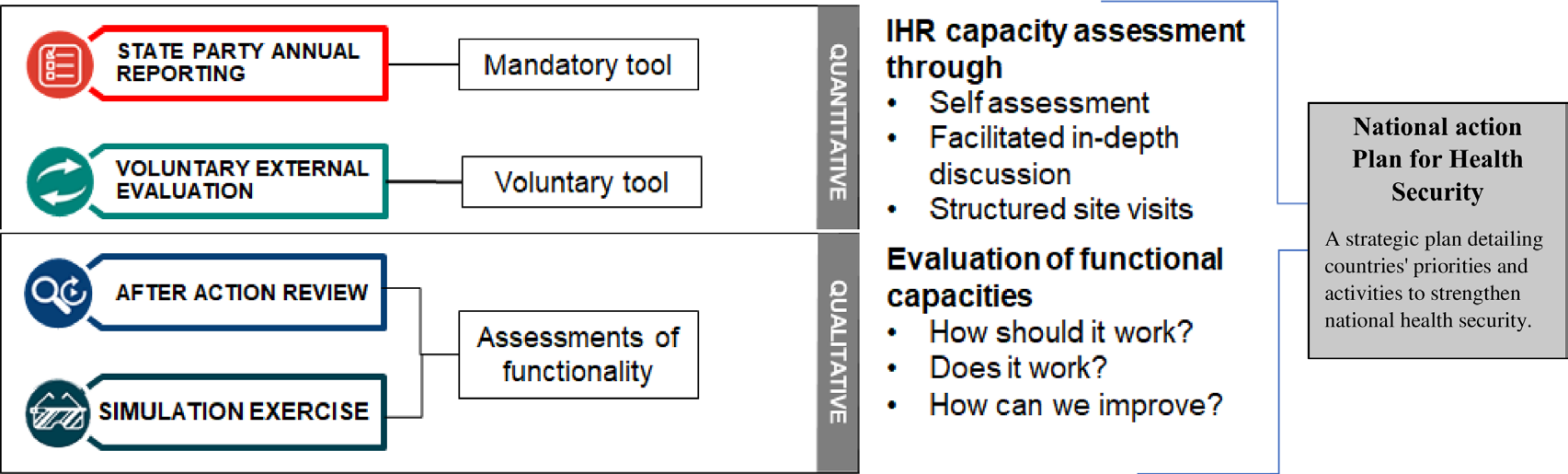
International Health Regulation Monitoring and Evaluation Framework (IHR MEF) tools by the WHO.

Thus, WHO EMR worked closely, from 2016 to 2023, with IHR National Focal Points in EMR Member States in order to promote the IHR MEF tools and evaluate closely the IHR core capacities implementation in the region. The following tools were used (figure 2):

### IHR State Party self-assessment annual report

SPAR is a mandatory tool for annual self-assessment, focusing on the 15 IHR core capacities, which member states must maintain to implement the IHR (2005). The SPAR tool includes 35 indicators that allow countries to assess their capacity to detect, assess, notify and respond to public health risks. In this study, SPAR data were collected from all 22 EMR member states from 2016 to 2023.^28^ It is the only mandatory tool among the IHR MEF. All states parties are required to have or develop and maintain minimum core public health capacities to implement the IHR (2005), and report the status of implementation annually, as stipulated in Article 54 of the Regulations (Reporting and review).^29^

Method of application: member states used SPAR to perform a structured self-assessment each year. The collected data were submitted via WHO’s web-based platform, e-SPAR, which facilitated the evaluation of national capacities and promoted transparency and accountability. In this study, we analysed annual SPAR data to observe trends in capacity building, with a focus on attributes related to laboratory systems, surveillance and emergency response, which are crucial for pandemic preparedness.^30^

#### Joint external evaluation

The JEE is part of the IHR MEF and, unlike the SPAR, is a voluntary, multisectoral process to assess the country’s capacity to prevent, detect and rapidly respond to public health risks, whether occurring naturally or due to deliberate or accidental events.^31^ The JEE allows countries to identify the major gaps within their health security system; to prioritise opportunities for enhanced preparedness, detection and response capacity, including setting national priorities and to allocate resources based on the findings.^31^

Since 2016, three editions of the tool were developed and published, the first in February 2016, the second in January 2018 and the third in June 2022. This last edition was updated and reviewed in order to measure the global health security, based on the feedback received related to the gaps shown during the response to the COVID-19 pandemic and other global health threats. The revised version of the JEE includes 19 capacities with previous and new technical areas from the previous two editions and a total of 56 indicators.^31 32^

The 19 technical areas in the third edition of the JEE tool are divided into four main groups including, prevent group with legal instruments, financing, IHR coordination, national IHR focal point functions and advocacy, antimicrobial resistance (AMR), zoonotic disease, food safety, biosafety and biosecurity and immunisation, as eight technical areas. Under the group detect, there are three technical areas, national laboratory system, surveillance and human resources. While under the respond group, there are five technical areas including, health emergency management, linking public health and security authorities, health services provision, infection prevention and control (IPC) and RCCE.^32^

The last group of technical areas is called the IHR Related Hazards and Points of Entry (PoEs) and Border Health, and it includes three important and politically sensitive technical areas, PoE and border health, chemical events and radiation emergencies.^32^ The recommendations coming out from the JEE to address the gaps of the health system in the country are the major source used to develop the National Action Plan for Health Security (NAPHS).

Method of application: JEE was conducted in EMR member states using a multisectoral approach, where WHO teams collaborated with national focal points and external experts. The evaluation process involved document reviews, working groups per technical area and site visits to verify the implementation of the IHR core capacities. The scoring system ranged from 1 (no capacity) to 5 (sustainable capacity), offering a structured method to assess preparedness levels.

#### National action plan for health security

NAPHS is a country-owned, multiyear, planning process based on One Health for all hazards and a whole-of-government approach. It captures national priorities for health security including key actions for addressing capacity gaps and resources required to accelerate the development of IHR core capacities.^33^ NAPHS covers the same 19 technical areas as the JEE, converting the JEE recommendations into activities in the form of an action plan using other assessments such as AAR/IAR, the Strategic Tool for Assessing Risks (STAR) tool, simulation exercise (SimEx) and others.

Since 2016, the member states and partners in EMR worked closely to support the development and implementation of NAPHS throughout the region using the needed technical instruments and advocacy to capture national priorities for health security including key actions for addressing capacity gaps, and resources required to accelerate the development of IHR core capacities, while supporting the fund-raising process to improve health security in the EMR.

The Pandemic Fund, officially known as the ‘Pandemic Emergency Financing Facility’, a collaborative partnership among donor governments, co-investor countries, foundations, civil society organisations and international agencies, was created in 2022 to provide a dedicated stream of additional, long-term funding for critical pandemic prevention, preparedness, and response in eligible low- and middle-income countries, through investments and technical support. It was established to provide a rapid financial response to countries facing the risk of pandemics, aiming to bridge the gap in global financing for pandemic response and strengthen the global health security architecture. The direct link between the Pandemic Fund and NAPHS lies in their shared objective of enhancing countries’ capacities to manage health emergencies.

The financial resources and support provided by the Pandemic Fund can be instrumental in implementing the NAPHS, facilitating investments in crucial areas such as surveillance, laboratory systems, workforce development and emergency response operations. By doing so, the Fund not only contributes to mitigating the immediate impacts of pandemics but also supports the long-term strengthening of health systems and preparedness measures outlined in the NAPHS, demonstrating a strategic synergy between global financial mechanisms and national health security efforts.

### Universal health and preparedness review

The Universal Health and Preparedness Review (UHPR) is a voluntary, transparent, member state-led peer review mechanism, aiming to establish an intergovernmental dialogue among member states to strengthen their respective national capacities for health emergencies and disaster preparedness, as well as to achieve the sustainability of cooperation and funding for such purposes.^34^ The UHPR is one of the 10 proposals presented by the director at the 75th World Health Assembly to strengthen the global architecture for health emergency preparedness and response (HEPR), as a key mechanism to ensure accountability among member states.^35^

Furthermore, the UHPR joins ongoing initiatives, such as the pandemic preparedness fund, the pandemic accord (treaty) and the amendments to the IHR, among others. WHO supported the Government of the Republic of Iraq to launch the pilot phase, as the first country in the EMR and the second globally, to conduct the UHPR in Iraq between December 2021 and March 2022.

Method of application: UHPR is mainly based on three stages. The Pre-Mission review when the country conducts a self-assessment of its health system’s preparedness and response capacities using predefined criteria to develop a comprehensive report. The High-level Mission where the mission report is endorsed by the government of the country and the peer-review process to evaluate the self-assessment country report, engage in dialogue with national authorities, and participate in structured workshop where there is exchange of information, evaluation of preparedness capacities, and offering recommendations for improvements with other nations.

### After-action review/ intra-action review

An AAR is a qualitative review of actions that are taken to respond to an emergency or a public health event to identify best practices and areas for improvement to be better prepared for the future events. It helps to assess the functionality of national capacities for preparedness and response to health emergencies.^36^ Considering the special nature of the COVID-19 pandemic, WHO developed the guidance for conducting a country COVID-19 IAR; thus, the countries have the needed tool to conduct periodic reviews during the event before its end, so they can continue to reflect on the ongoing response and revise national and subnational response strategies and plans as needed.^37^

Both AAR and IAR tools were used to support EMR member states to identify best practices, gaps and lessons learnt from the response to different events and allow stakeholders of the COVID-19 response to review the functional capacities of public health and emergency response systems at the national or subnational levels to propose corrective measures and actions for immediate remediation or sustained improvement of the COVID-19 outbreak response.^36 37^

Method of application: AAR was applied following major public health events such as the COVID-19 pandemic and outbreaks of diseases like cholera in Somalia and diphtheria in Pakistan. The review processes were facilitated through stakeholder workshops, where health officials, responders and community representatives shared experiences and identified best practices and areas needing improvement. The IAR was implemented in several EMR countries during the COVID-19 pandemic. National health authorities conducted mid-response reviews, focusing on aspects like case management, public health communication and contact tracing. The IAR process involved key informant interviews and in-person working groups, where feedback from multiple stakeholders was integrated into real-time policy adjustments.

### Simulation exercises

The SimEx can help develop, assess and test functional capabilities of emergency systems, procedures and mechanisms to allow to respond properly to outbreaks or public health emergencies. It simulates an emergency situation to which a described or simulated response is made. WHO defines four types of exercises, including the discussion-based tabletop exercises (TTXs), as well as the operations-based exercises (drills, functional exercises and field/full-scale exercises).^38^

Method of application: SimEx was widely used in EMR countries to test the preparedness and response capacities outlined in their NAPHS. WHO defines four types of exercises: TTXs, drills, functional exercises and full-scale exercises. This study focused on the TTXs and functional exercises conducted in different member states.

### Ethics approval

No ethical approval was required for this study.

## Results

Using the IHR MEF tools, all EMR member states (22 countries, namely, Afghanistan, Bahrain, Djibouti, Egypt, Iran (Islamic Republic of), Iraq, Jordan, Kuwait, Lebanon, Libya, Morocco, Oman, Palestine, Pakistan, Qatar, Saudi Arabia, Somalia, Sudan, the Syrian Arab Republic, Tunisia, UAE, Yemen) succeeded to complete the SPAR during the past 2 years, since the launching of the SPAR updated version, in 2021 (figure 1).

**Figure 3:**
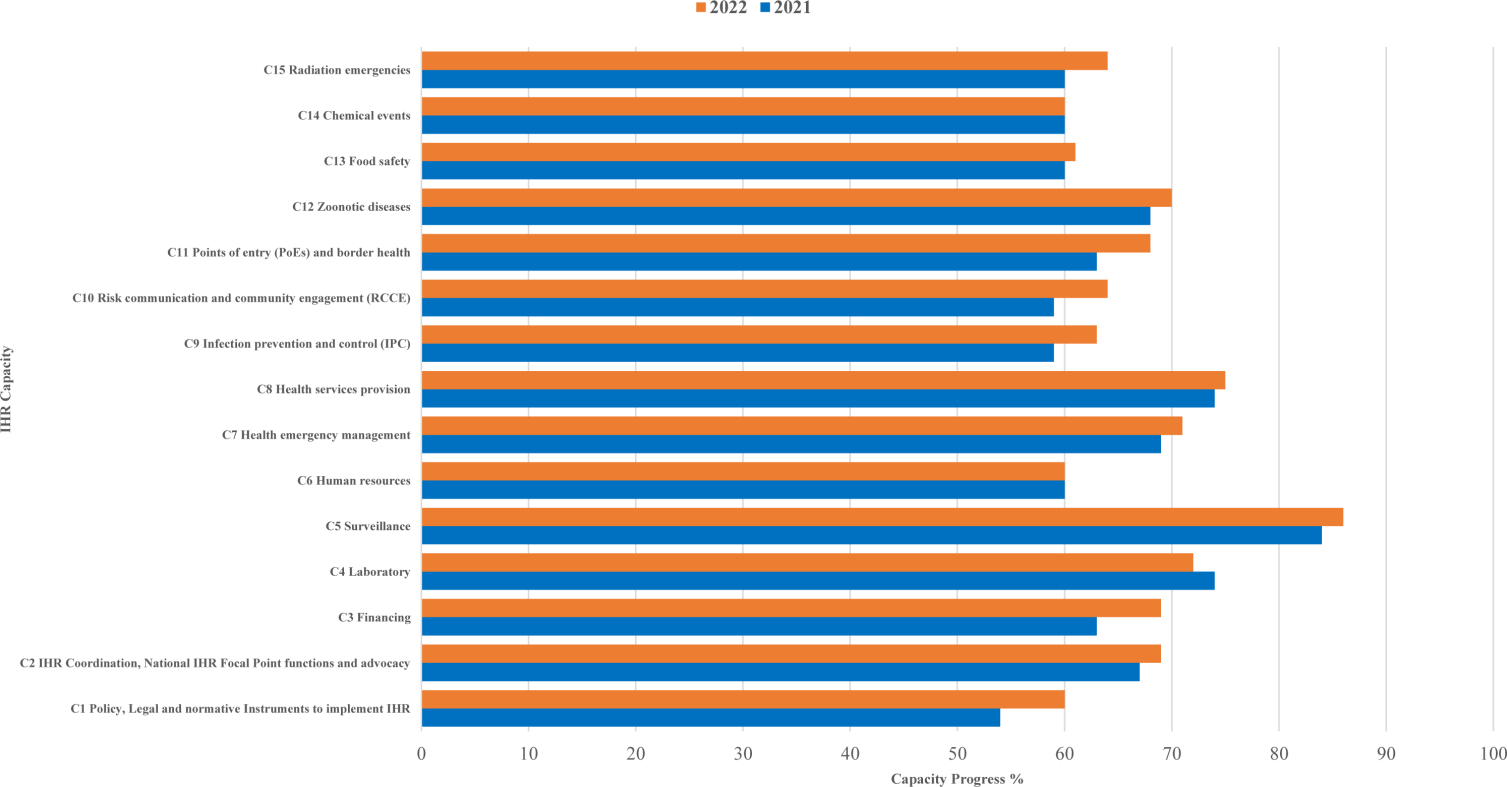
IHR core capacities progress in EMR, according to SPAR 2021 and 2022 (n=21 countries).

The IHR core capacities with the highest scores were ‘C5 Surveillance’ with a score of 84% in 2021 and 86% in 2022, ‘C8 Health services provision’ with a score of 74% in 2021 and 75% in 2022 and ‘C4 Laboratory’ with a score of 74% in 2021 and 72% in 2022; while the core capacity with the lowest score was ‘C1 Policy, Legal and normative Instruments to implement IHR’ with a score of 54% in 2021 and 60% in 2022 (figure 3).

Since 2016, when the first edition of the JEE was launched, up to August 2023, 20 EMR member states have completed the first round of the JEE in the region (namely, Afghanistan, Bahrain, Djibouti, Egypt, Iraq, Jordan, Kuwait, Lebanon, Libya, Morocco, Oman, Pakistan, Qatar, Saudi Arabia, Somalia, Sudan, the Syrian Arab Republic, Tunisia, UAE, Yemen). With the first JEE conducted in the region, in April 2016, in Pakistan and the last JEE completed, in August 2023, in Sanaa (Yemen). EMR member states have already started their second round of JEE, with two member states, Pakistan and Iraq, being the first countries in the region to complete the evaluation for the second time, in May and September 2023, respectively, while several countries started the process to be completed during the upcoming few months.

The EMR is one of the highly ranked, among the six WHO regions, in terms of completion of the JEE globally, with a 91% completion ratio (20 completed external evaluation missions out of 22 countries with 17 published reports online and two in the pipeline), and it comes second after the African region with a completion ratio of 94%. Among the 20 countries that completed the first round of JEE in the EMR, the UAE, Oman, Egypt, Bahrain and Saudi Arabia scored among the highest capacities score average and had the highest average score in each technical group with Prevent, Detect, Respond and PoEs and IHR hazards, with small differences in the scores from other member states including Kuwait, Qatar and Morocco. The regional average score for the different technical groups were 53% average score (mean) for ‘Prevent’ group, 58% average score for ‘Detect’ group, 58% average score for ‘Respond’ group and 52% average score for PoEs and IHR hazards (n=20) (table 1).

**Table 1:** JEE capacities score average among EMR member states (alphabetical order) and the dates of completion of the first round between 2016 and 2023

State party	JEE date completed	Technical group	Regional average
Afghanistan	December 2016	Prevent	53%
Bahrain	September 2016		
Djibouti	July 2018		
Egypt	September–October 2018		
Iraq	March 2019		
Jordan	August–September 2016	Detect	58%
Kuwait	May 2017		
Lebanon	July 2016		
Libya	July 2018		
Morocco	June 2016		
Oman	April 2017	Respond	58%
Pakistan	April–May 2016		
Qatar	May–June 2016		
Saudi Arabia	March 2017		
Somalia	September 2016		
Sudan	October 2016	PoEs and IHR hazards	52%
Syrian Arab Republic	June 2023		
Tunisia	December 2016		
UAE	March 2017		
Yemen	May–June 2023 (Aden)August 2023 (Sanaa)		

EMR, Eastern Mediterranean Region; IHR, International Health Regulations; JEE, Joint External Evaluation; PoEs, Points of Entry.

The member states that conducted the JEE were able to develop their NAPHS using the recommendations of the JEE to develop activities allowing them to respond to the gaps in their health system. The report of the twentieth developed NAPHS in the region, for Yemen, is currently under final review.

Two member states in the EMR have already gone through a second round of revision and update of their national plans in 2022, namely Tunisia and Sudan. EMRO is following the WHO Strategy (2022–2026) for the NAPHS^39^ to support member states to accelerate the development, implementation, monitoring and revision of their NAPHS from 2022 to 2026.

Great efforts were spent by the Government of Iraq, during the Pre-UHPR Mission (phase 1) and the High-level Mission (phase 2), to collect and review all health background documents of the country, to conduct several meetings at the national and Governorate levels between December 2021 and February 2022 in order to engage in discussions, collect data and develop risk profiles for different sectors in the country. Field visits were made to seven governorates, namely, Baghdad, Al-Anbar, Nineveh, Basra, Erbil, Sulaymaniyah and Duhok in Kurdistan Region. The final report of the UHPR Iraq pilot phase was published on the WHO website in April 2023.^40^

The AAR tool was used after several events in different countries in the region, including infectious diseases, natural disasters and mass gathering events. Since 2018, five countries, namely, Iran (Islamic Republic of), Morocco, Pakistan, Qatar and Sudan, used the tool to assess or identify what worked well or did not and how these practices can be maintained, improved, institutionalised and shared with relevant stakeholders.

Since the COVID-19 emergence in the region in January 2020, the IAR tool was used by 12 countries, namely, Afghanistan, Bahrain, Egypt, Jordan, Kuwait, Lebanon, Pakistan, Saudi Arabia, Somalia, the Syrian Arab Republic (three times), Sudan and Tunisia (twice), in order to review the functional capacities of public health and emergency response systems at the country level to identify best practices, gaps and lessons learnt and propose corrective measures and actions for immediate improvement of the COVID-19 response.

Since 2017 up to December 2023, 19 EMR countries used different types of SimEx in order to review, assess and/or test the procedures, operational plans, guidelines and standard operating procedures developed for a better preparedness to respond properly during the upcoming events. The following countries were supported by EMRO, including Afghanistan, Bahrain, Djibouti, Egypt, Iran (Islamic Republic Of), Iraq, Jordan, Kuwait, Lebanon, Libya, Morocco, Oman, Pakistan, Palestine, Qatar, Somalia, Sudan, Tunisia and UAE, to technically conduct different SimEx.

As part of the UHPR process, the conduction and facilitation of two TTXs were done, one at the central level in Baghdad and the second in the Kurdistan Region in Erbil. Different ministries that played a major role during the response to COVID-19 in the country participated in multisectoral exercises. The TTXs were conducted to assess the functionality of Iraq’s HEPR system and its components, identify areas of strengths and weaknesses and review some of the country’s planning assumptions.^40^

## Discussion

The countries in the EMR have taken a number of steps in track to leverage their health security and build better capacities for emergency preparedness during the past few years. The data of our study shows that in member states such as Egypt and Iraq, COVID-19 triggered a reassessment of health emergency preparedness, with significant improvements observed in laboratory capacities and risk communication, as reflected in their SPAR scores between 2019 and 2022. This demonstrates that the pandemic acted as a catalyst for strengthening national health security systems.

The integration of the One Health approach was obvious in member states like Saudi Arabia, where enhanced coordination between the three sectors: human, animal and environmental health was reflected in increased JEE scores for zoonotic disease preparedness. These findings align with improvements in the national laboratory system, surveillance and antimicrobial resistance (AMR) control. The importance of the One Health approach in health emergency preparedness is significant through prevention, early detection and rapid response to zoonotic diseases and is directly linked to other technical areas including food safety, national laboratory systems, surveillance and AMR.^41^

Another approach for leveraging health security and emergency preparedness is RCCE. RCCE became an essential public health intervention that is key to successful emergency response throughout the prevention, preparedness, response and recovery phases.^42^ Our analysis of the COVID-19 response in Jordan and Pakistan revealed that countries which implemented strong RCCE programmes witnessed improved public adherence to preventive measures, reflected in reduced case fatality rates and enhanced public trust, as documented in the AARs conducted in 2021 and 2022. It enables tailored, targeted and more efficient emergency interventions that build trust between people and health authorities, address rumours, infodemics and misinformation, lead to the acceptance and uptake of protective measures and ultimately, save lives. This was clearly demonstrated during the COVID-19 pandemic response and the monkeypox (Mpox) outbreak in 2022, declared as the Public Health Emergency of International Concern (PHEIC) by the WHO Director General on 27 July 2022,^43^ before declaring the end of Mpox as PHEIC on 11 May 2023.^44^

PoEs and border health play a critical role in health emergency preparedness and this was reflected in the IHR (2005) from Article 19 ‘General obligations’ at the PoE to Article 23 ‘Health measures on arrival and departure’.^29^ Data from the JEE assessments in Egypt and Jordan indicated that improvements in PoEs preparedness, such as the implementation of cross-border surveillance and rapid response measures, were critical in preventing cross-border transmission of diseases, particularly during the COVID-19 pandemic. These measures are meant to prevent the international spread of diseases and include cross-border collaboration in different sectors including surveillance, reporting, notifications and trainings and SimExs and others.

The IHR MEF tools were helpful in advancing health emergency preparedness across the EMR. Our analysis of SPAR data from 2016 to 2023 shows that 100% of member states completed the annual self-assessments, with notable improvements in surveillance and laboratory capacities. This progress was most evident in Gulf Cooperation Council (GCC) countries, which achieved the highest scores in the region. Our findings from the second round of JEE conducted in Pakistan and Iraq in 2023 showed that both countries made significant progress in filling the gaps identified in the first round. Notable improvements were seen in IPC and health services provision, demonstrating the effectiveness of the NAPHS implemented after the first JEE.

The Pandemic Fund finances critical investments to strengthen pandemic prevention, preparedness and response capacities at national, regional and global levels, with a focus on low- and middle-income countries.^45^ This fund serves as a vital investment, especially for the EMR, where NAPHS is not adequately financed, channelling the much-needed resources to bolster pandemic prevention, preparedness and response capacities at national, regional and global levels. With the technical support from partners led by WHO, 14 countries in the EMR managed to submit their applications for the first round of the Call for Proposals in May 2023. Two countries, Palestine and Yemen, succeeded in securing funding and the rest of the countries are getting prepared for the second round of submission of their proposals in quarter one of 2024.

The UHPR pilot in Iraq provided valuable data on health security gaps and best practices. Our analysis revealed that the establishment of crisis management cells at the Governorate level, as well as Iraq’s 2021 Health Insurance Law, were important in advancing Universal Health Coverage. However, gaps in emergency preparedness, particularly related to political instability, continue to hinder the full implementation of the UHPR recommendations.^40^ While the major gaps included limited understanding and practice of emergency preparedness and the Incident Management System in the country. Finally, the major challenges faced by the health system in Iraq were the volatile political, social, economic and security setting compounded by a protracted military, political, economic, humanitarian and security crisis.^40^ The same challenges are faced by more than half of the EMR countries.^46^

In November 2022, Egypt hosted the 2022 United Nations Climate Change Conference (COP-27), held in Sharm El-Sheikh, and a workshop was conducted in October 2022 to evaluate the preparedness plan of the country for this huge event. During the study, we analysed the AAR report developed following the Fédération Internationale de Football Association (FIFA) World Cup 2022 in Qatar, where improvements in mass gathering preparedness were observed, particularly in health service provision and cross-sectoral coordination. This was reflected in the successful management of public health risks during the event, as documented in the review.

These activities not only strengthened the region’s ability to handle large-scale events but also provided invaluable insights and lessons for future planning putting into consideration that the region is host to two of the largest religious gatherings globally, Pilgrimage (Haj) in Mecca, Saudi Arabia, estimated to be more than 2.5 million pilgrims in 2023^47^ and Arba'een in Karbala, Iraq, estimated by 21 million pilgrims in 2022.^48^

Believing that the commitment to preparedness extends beyond specific events, the WHO Regional Office organised workshops to assess the readiness of member states for a wide range of health emergencies and threats. Data from the IHR Capacity Building Workshop in Somalia and the SimEx in Bahrain in 2023 showed measurable improvements in emergency preparedness capacities, particularly in surveillance and rapid response. These activities resulted in enhanced preparedness for future public health emergencies, as evidenced by increased scores in the JEE follow-up assessments.

The global COVID-19 pandemic profoundly affected societies, health systems and economies worldwide. In response, the WHO Regional Office for the Eastern Mediterranean conducted an independent review to evaluate the pandemic response in the EMR. The Dalberg report recommended transforming the temporary capacities built during the COVID-19 response into permanent structures, strategies and networks. Following this, EMRO undertook a mapping of gains initiative to sustain the progress made. The region, comprising 22 countries and territories, including nine fragile and conflict-affected states, embarked on this initiative to document new capacities and understand effective coordination and collaboration. The comprehensive exercise involved a desk review, expert interviews and an online survey administered across all member states, culminating in rich data collection and analysis focused on five core areas: Emergency Coordination, Collaborative Surveillance, Access to Countermeasures, Safe and Scalable Care and Community Protection. Despite some limitations, such as incomplete data and staff turnover, the review provides detailed findings on key gains, new capacities and sustainability priorities, aiming to bolster future preparedness and health security in the EMR.^49^

The collective efforts of member states, supported by WHO and regional partners, have resulted in measurable improvements in health security, as demonstrated by the increased completion of SPAR and JEE evaluations across the region. These findings show and emphasise on the importance of continued investment in capacity-building activities and cross-sectoral collaboration to sustain the gains made in health emergency preparedness.

### Limitations

The presence of six countries in the region classified as high-income countries, the GCC countries, and three politically stable countries including Egypt, Jordan and Morocco, may have caused the data to be skewed based on the high capacities in these countries compared with the rest of the region.

## Conclusion

In conclusion, WHO EMR worked hard to progress in strengthening health security and enhancing emergency preparedness capacities over recent years. The region needs more advocacy for the IHR core capacity implementation at the highest official level, more empowerment for the IHR National Focal Points (NFPs) and IHR Review Committee to play as a champion for IHR globally. There is a need for continuous capacity building for the IHR NFPs, and it is important to highlight the added value of the partnership with different stakeholders including Academia.

Leveraging health security and building preparedness capacities will support EMR to be better equipped to mitigate the impact of upcoming health emergencies. While the EMR took steps towards achieving its health security goals, the pathway is still long, the region still faces huge challenges. It necessitates a collective commitment and transparency from member states, international organisations, civil society and the private sector to overcome challenges, build resilient health systems and ensure a safer and healthier future for all.

## Data Availability

Data are available in a public, open access repository.
